# Detrimental effects of COVID-19 in the brain and therapeutic options for long COVID: The role of Epstein–Barr virus and the gut–brain axis

**DOI:** 10.1038/s41380-023-02161-5

**Published:** 2023-07-04

**Authors:** Kenji Hashimoto

**Affiliations:** Division of Clinical Neuroscience, Chiba University Center for Forensic Mental Health, Chiba, 260-8670 Japan

**Keywords:** Depression, Neuroscience

## Abstract

The coronavirus disease 2019 (COVID-19) pandemic caused by severe acute respiratory syndrome coronavirus 2 (SARS-CoV-2) infection has resulted in a serious public health burden worldwide. In addition to respiratory, heart, and gastrointestinal symptoms, patients infected with SARS-CoV-2 experience a number of persistent neurological and psychiatric symptoms, known as long COVID or “brain fog”. Studies of autopsy samples from patients who died from COVID-19 detected SARS-CoV-2 in the brain. Furthermore, increasing evidence shows that Epstein–Barr virus (EBV) reactivation after SARS-CoV-2 infection might play a role in long COVID symptoms. Moreover, alterations in the microbiome after SARS-CoV-2 infection might contribute to acute and long COVID symptoms. In this article, the author reviews the detrimental effects of COVID-19 on the brain, and the biological mechanisms (e.g., EBV reactivation, and changes in the gut, nasal, oral, or lung microbiomes) underlying long COVID. In addition, the author discusses potential therapeutic approaches based on the gut–brain axis, including plant-based diet, probiotics and prebiotics, fecal microbiota transplantation, and vagus nerve stimulation, and sigma-1 receptor agonist fluvoxamine.

## Introduction

**Fig. 1:**
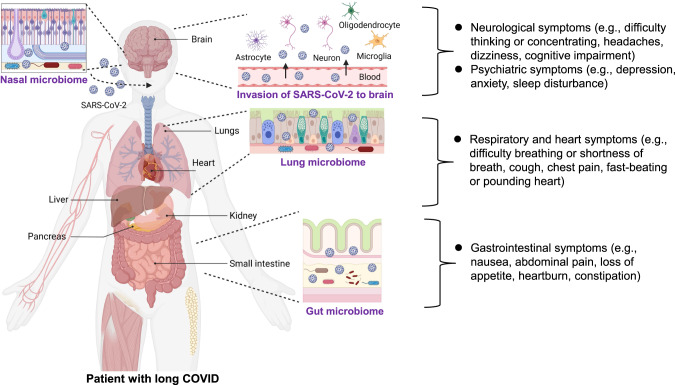
Long-term sequelae in COVID-19 survivors. Coronaviruses, such as SARS-CoV-2, can enter the brain through a direct infection pathway (e.g., blood circulation and neuronal pathways), and other mechanisms. Studies using autopsies from patients who died from COVID-19 detected SARS-CoV-2 in the brain. The ability of SARS-CoV-2 to enter the brain implies its role in the long-term neurological symptoms (e.g., difficulty thinking or concentrating, headaches, dizziness, cognitive impairment) and psychiatric symptoms (e.g., depression, anxiety, sleep problems) observed in COVID-19 survivors. The other most common symptoms of long COVID were general symptoms (e.g., loss of smell or taste, tiredness or fatigue), respiratory and heart symptoms (e.g., difficulty breathing or shortness of breath, cough, chest pain, fast-beating or pounding heart), and gastrointestinal symptoms (e.g., nausea, abdominal pain, loss of appetite, heartburn, constipation). There is also increasing evidence that alterations in the gut, nasal, oral, and lung microbiomes may contribute to long COVID symptoms. Part of the figure was designed using resources from Biorender.com.

Coronavirus disease 2019 (COVID-19), caused by infection with severe acute respiratory syndrome coronavirus 2 (SARS-CoV-2), continues to threaten public health worldwide. COVID-19 patients frequently experience sequelae such as shortness of breath or dyspnea, and fatigue or exhaustion [1–3]. A number of neurological and psychiatric symptoms have also been linked to COVID-19, including anosmia (or loss of smell), ageusia (or loss of taste), cognitive impairment, depression, anxiety, and sleep disturbance [1–3]. Long-lasting symptoms following SARS-CoV-2 infection are known as long COVID or post-COVID conditions [4–6]. The World Health Organization reported that long COVID is defined as the symptoms that usually occur 3 months after infection of SARS-CoV-2, with symptoms lasting for at least 2 months [7]. Long COVID has impacts on multiple tissues, including respiratory and non-respiratory organs such as the heart, kidney, immune system, pancreas, gastrointestinal (GI) tract, and brain (Fig. 1) [3, 6, 8]. However, the precise biological mechanisms underlying long COVID remain elusive. At present, there are several hypothesized mechanisms, including immune dysregulation, dysbiosis of the gut microbiome, autoimmunity, clotting and endothelial abnormalities, and altered neurological signaling [3]. Unfortunately, current diagnostic and treatment options are insufficient.

In this article, the author reviews the detrimental effects of COVID-19 on the brain, and the underlying mechanisms and therapeutic potential for long COVID.

## Neurological and psychiatric symptoms in long COVID patients

Although COVID-19 was initially recognized as a severe respiratory disease, SARS-CoV-2 also has an impact on non-respiratory organs, including the brain. In addition to general symptoms (i.e., fatigue or tiredness) and heart and respiratory symptoms (i.e., shortness of breath or difficulty breathing, chest pain, cough, pounding heart, or fast-beating), there are a variety of psychiatric symptoms (i.e., anxiety, depression, sleep disturbance) and neurological symptoms (i.e., concentrating or difficulty thinking, dizziness, headaches, cognitive impairment) (Fig. 1) [3]. A systematic review of COVID-19 survivors (*n* = 250,351) reported that the proportion of COVID-19 survivors experiencing at least one symptom of long COVID was 54% at 6 months or more [9]. A meta-analysis (*n* = 81) showed a significant increase in the number of subjects experiencing fatigue and/or cognitive impairment following the resolution of acute symptoms in COVID-19 patients [10]. A recent meta-analysis using a large sample (*n* = 1,285,407) from 32 countries reported that approximately half of COVID-19 survivors had persistent symptoms in the 12 months after hospital discharge [11]. Unfortunately, there are currently no potential therapeutic drugs for long-term neurological and psychiatric symptoms in COVID-19 survivors.

## Impact of SARS-CoV-2 on the brain

### Detection of SARS-CoV-2 in the brain of patients who died from COVID-19

Increasing evidence shows that SARS-CoV-2 is detectable in the brain of patients who died from COVID-19. In May 2020, Puelles et al. [12] reported the SARS-CoV-2 viral load in the autopsy tissues from patients who died from COVID-19. High copy numbers of SARS-CoV-2 per cell were detected in the lungs, and lower copy numbers of virus were detected in the brain, indicating the broad organotropism of SARS-CoV-2 in the human body [12]. Another study reported low copy numbers of virus in the postmortem brains of patients who died from COVID-19 [13]. Furthermore, Song et al. [14] reported specific staining of spike protein in the brains of patients (*n* = 3) who died from severe COVID-19, and pathological changes in the autopsied brains were associated with infection but minimal immune cell infiltration [14]. A recent study on autopsied COVID-19 patients showed that SARS-CoV-2 was widely distributed in a number of respiratory and non-respiratory organs, including the brain. Interestingly, in their study, few histopathological changes were detected in the brain, despite a substantial viral burden [15]. Collectively, these findings show the likelihood that SARS-CoV-2 can enter the brain after infection, resulting in multiple persistent neurological and psychiatric symptoms [16–18]. A recent study using postmortem brain samples demonstrated similar neuropathological features in the brain from patients with delta, omicron, and non-delta/non-omicron variants of SARS-CoV-2 [19], suggesting common neuropathological mechanisms of SARS-CoV-2 variants. It is reported that the spike protein, which is derived by SARS-CoV-2 infection and generated by SARS-CoV-2 vaccines, could be able to cross the blood–brain barrier (BBB), resulting in blood clots and neuroinflammation in the human brain [20].

### Evidence from cell culture assays and rodents

A study using an in vitro BBB cell culture assay showed that the spike protein S1 can cross the human brain endothelial cell barrier [21]. Intranasal or intravenous administration of radioiodinated S1 protein of SARS-CoV-2 could cross the BBB in mice by adsorptive transcytosis [22]. SARS-CoV-2 is reported to cross the BBB via a transcellular pathway accompanied by basement membrane disruption but no alteration of tight junctions [23]. Furthermore, Song et al. [14] showed that SARS-CoV-2 caused significant neuronal death in the human brain organoids. Using mice overexpressing human ACE2, they confirmed increasing viral titers in the mouse brain after intranasal administration of SARS-CoV-2. Interestingly, lung hACE2-expressing mice showed signs of lung pathology after intranasal administration of SARS-CoV-2, but no weight loss or death. By contrast, brain hACE2-expressing mice showed weight loss and death after intraventricular administration of SARS-CoV-2. These data strongly highlight the neuroreplicative potential and lethal consequences of SARS-CoV-2 infection in the brain [14]. Collectively, it seems that SARS-CoV-2 infection in the brain might contribute to severe symptoms in patients with COVID-19, resulting in high mortality.

### Brain imaging data of COVID-19 patients

There is increasing evidence showing abnormalities in the brain function of COVID-19 survivors. The presence of leptomeningeal enhancement or hyperintense lesions in the brains of COVID-19 patients is associated with an increased viral load in the cerebrospinal fluid [24]. A brain imaging study using the United Kingdom Biobank (*n* = 785) demonstrated that long-lasting detrimental effects linked to the olfactory cortex after SARS-CoV-2 infection may be associated with the earliest and most common symptoms (e.g., loss of taste and smell) in COVID-19 survivors [25, 26]. Taken together, these findings indicate that brain structure alterations likely contribute to long COVID symptoms [26, 27].

### Effects of intranasal COVID-19 vaccines on the brain and nasal microbiome

Intranasal administration of COVID-19 vaccine has several advantages over conventional intramuscular injection because the nasal mucosa is the initial site of infection [28–31]. Many nasal vaccines for COVID-19 are under development worldwide.

Drugs administered via intranasal delivery can cross the BBB, leading to the direct delivery of the drug to the brain. The intranasal route has been increasingly used to deliver drug candidates to the brain in the treatment of a number of neurological and psychiatric disorders [32]. The intranasal route offers the following advantages: (1) candidate drugs are delivered directly to the brain via the BBB; (2) first-pass metabolism of the drug candidate in the liver is avoided; and (3) the onset of action of the drug candidate in the brain is delayed [32]. Mucosal immunity induced following the injection of intranasal vaccines is not limited to local respiratory sites [31]. However, it is currently unclear whether COVID-19 vaccines injected intranasally can enter the human brain, and further studies are needed. Considering the abundance of nasal microbes in the membranes of the nasal mucosa (Fig. 1), it may also be interesting to examine the effects of nasal COVID-19 vaccines on the nasal microbiome.

## Underlying mechanisms of long COVID

The precise biological mechanisms underlying long COVID remain elusive; however, several hypotheses have been proposed. These hypotheses include persistent reservoirs of SARS-CoV-2 in tissues, dysregulation of the immune system with or without reactivation of pathogens such as Epstein–Barr virus (EBV), dysbiosis of the gut microbiome, autoimmunity and priming of the immune system from molecular mimicry, microvascular blood clotting with endothelial dysfunction, and dysfunction of body–brain communication with or without involvement of the vagus nerve [3].

### Reactivation of Epstein–Barr virus (EBV)

**Fig. 2:**
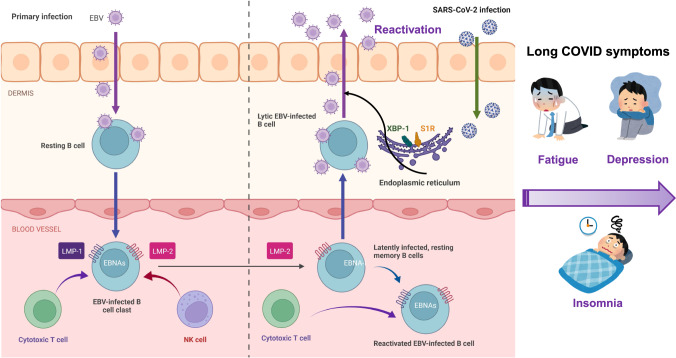
Possible role of EBV reactivation in long COVID. EBV directly infects resting B cells or epithelial cells, and is then stored in the infected memory B cells of the peripheral blood that express latent membrane protein 2 (LMP-2) and EBV nuclear antigens (EBNAs). After SARS-CoV-2 infection, these B cells can cause EBV reactivation, resulting in severe systemic inflammation. As the interaction of the XBP-1 (X-box binding protein 1) with S1R (sigma-1 receptor) may play a role in EBV reactivation, sigma-1 receptor agonists (e.g., fluvoxamine) may attenuate EBV reactivation, resulting in reduced long COVID symptoms. Part of the figure was designed using resources from Biorender.com and www.irasutoya.com.

EBV is a double-stranded DNA virus belonging to the herpes family, which is responsible for infectious mononucleosis. EBV is one of the most common viruses in humans. More than 90% of adults worldwide have been infected with EBV, and most infected individuals are asymptomatic. Furthermore, EBV has the ability to switch from a latent to a lytic state in response to a variety of stimuli such as infection and psychological stress (Fig. 2) [33, 34]. It is known that EBV directly infects resting B cells or infects epithelial cells. In the blood vessel, EBV is present in the infected memory B cells that express the latent membrane proteins (LMP-1 and LMP-2) and EBV nuclear antigens (EBNAs). During COVID-19 pandemic, SARS-CoV-2 infection could reactivate EBV-infected B cells that express LMP-2 and EBNA-1 (Fig. 2) [33]. EBV infection is also known to cause a number of central nervous system conditions such as viral meningitis, encephalitis, sleep disorders, psychosis, and multiple sclerosis [35–37].

A retrospective study from Wuhan University (China) was the first to show that subjects co-infected by SARS-CoV-2 and EBV have an approximate three-fold increased risk of severe symptoms compared with subjects infected with SARS-CoV-2 alone [38], suggesting that EBV reactivation may contribute to the severity of clinical symptoms after infection. Furthermore, Gold et al. [39] reported that many symptoms (e.g., fatigue, insomnia, headaches, myalgia, confusion) of long COVID may be due to EBV reactivation by SARS-CoV-2 infection. Additionally, a longitudinal study using COVID-19 patients (*n* = 309) demonstrated that EBV viremia is associated with long COVID symptoms such as fatigue and sputum [40]. Recently, Klein et al. [41] demonstrated that EBV and varicella-zoster virus, which causes chickenpox and shingles, might recently have been “reactivated”. Using a cohort (*n* = 280) with prior SARS-CoV-2 infection, Peluso et al. [42] reported that long COVID symptoms such as fatigue and cognitive impairment 4 months after infection were independently associated with recent reactivation of EBV. Collectively, it appears that a number of long COVID symptoms may be due to EBV reactivation induced by inflammation after SARS-CoV-2 infection (Fig. 2) [39, 43].

Currently, there are no therapeutic drugs to block EBV reactivation. It is reported that X-box-binding protein 1 (XBP-1) in the endoplasmic reticulum (ER) could activate EBV gene expression in combination with protein kinase D, suggesting an essential role of XBP-1 in EBV reactivation [44]. It is also shown that the ER chaperone sigma-1 receptor [45] could modulate the expression of ER stress-related proteins through the regulation of XBP1 [46, 47]. Given the role of the sigma-1 receptor on the regulation of XBP-1 in the ER [46, 47], it is proposed that the potent sigma-1 receptor agonist fluvoxamine [a selective serotonin reuptake inhibitor (SSRI)] may be a potential therapeutic drug for long COVID (Fig. 2) [48–53]. A recent population-based retrospective study reported that the use of SSRIs with sigma-1 receptor agonism at baseline (at or prior to COVID-19 infection) was associated with a reduced risk of long COVID [54]. A recent randomized placebo-controlled double-blind trial demonstrated that treatment with fluvoxamine (*n* = 42, 100 mg daily for 10 days) during active COVID-19 significantly reduced the incidence of fatigue 12 weeks after the primary COVID-19 compared with placebo group (*n* = 43) [55]. All other symptoms except poor concentration were less in the fluvoxamine group compared with placebo group although statistical analysis did not reach to significant differences because of low dose (100 mg daily) [55]. Therefore, it is of great interest to examine whether fluvoxamine (e.g., 100 mg twice daily for 10 days) could block the occurrence of long COVID symptoms [51–53]. A randomized placebo-controlled trial of fluvoxamine for long COVID (NCT05874037) is ongoing at Washington University (St. Louis, MO, USA).

## Abnormalities in the gut–microbiota–brain axis

### Dysbiosis of the gut microbiome in COVID-19 survivors

**Fig. 3:**
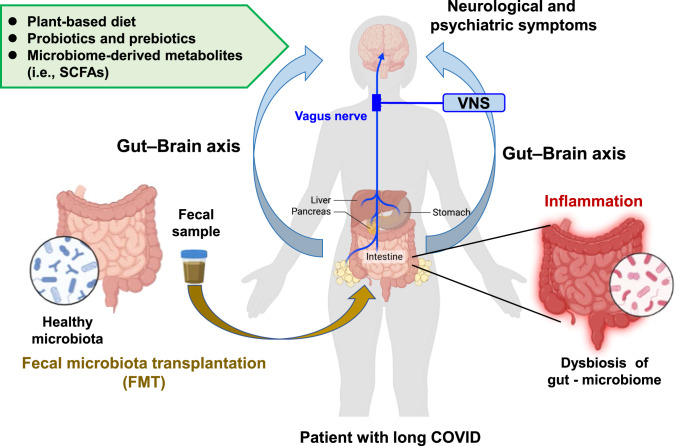
The gut–brain axis as a therapeutic target for long COVID. Patients with long COVID have persistent dysbiosis of the gut microbiome, resulting in a number of persistent GI symptoms, as well as neurological and psychiatric symptoms through the gut–brain axis. A plant-based diet, probiotics and prebiotics, and microbiome-derived metabolites [e.g., short-chain fatty acids (SCFAs: butyrate)] may have prophylactic or therapeutic potential for patients with long COVID. Given the role of the gut–microbiota–brain axis in neuroinflammation, fecal microbiota transplantation (FMT) from healthy controls may be a potential therapeutic tool for patients with long COVID. In addition, given the crucial role of the vagus nerve in the gut–brain axis, vagus nerve stimulation (VNS) could improve a number of neurological and psychiatric symptoms in patients with long COVID. This is a slight modification of a previously published figure [94]. Part of the figure was designed using resources from Biorender.com.

A number of patients have persistent GI symptoms (e.g., nausea, abdominal pain, loss of appetite, heartburn, constipation) after recovering from COVID-19 (Fig. 1) [56, 57]. Furthermore, there are increasing reports showing dysbiosis of the gut microbiome in COVID-19 patients (Fig. 3), although the mechanism by which SARS-CoV-2 causes dysbiosis of the gut microbiome remains unclear. One hypothesis is that when SARS-CoV-2 enters the lung it causes tissue damage via severe inflammation, resulting in a cytokine storm and dysbiosis. Another hypothesis is that SARS-CoV-2 can invade the intestine after infection, causing impairment of the intestinal structure and breakdown of the intestinal epithelial barrier, promoting intestinal inflammation and dysbiosis (Fig. 1) [58]. Importantly, SARS-CoV-2 was detected in the feces of COVID-19 patients [59], with virus being present in feces samples 4 months after infection [60].

Increasing evidence suggests that GI symptoms in COVID-19 survivors are associated with alteration of the gut microbiome [61–64]. Yeoh et al. [65] demonstrated that gut microbiome composition was significantly altered in COVID-19 patients (*n* = 100) compared with non-COVID-19 controls (*n* = 78), and that gut microbiome composition in hospitalized patients (*n* = 87) was associated with disease severity. Interesting, there were associations between the composition of the gut microbiome and the levels of inflammatory markers in the blood of COVID-19 patients. Furthermore, significant alterations in the gut microbiota composition remained evident in recovered patients (*n* = 27) compared with non-COVID-19 subjects. A recent meta-analysis of 16 studies showed that the diversity of the gut microbiome in COVID-19 patients in both the acute phase and the recovery phase was lower than that of non-COVID-19 subjects [66]. In addition, they found a reduction of anti-inflammatory butyrate-producing microbes and enrichment of taxa with pro-inflammatory effects in COVID-19 patients during the acute phase. Thus, dysbiosis of the gut microbiota persisted even after recovery from COVID-19 [66]. Collectively, it is likely that dysbiosis of the gut microbiome may contribute to persistent symptoms in COVID-19 survivors (Fig. 3).

Fecal metabolites in Chinese patients with COVID-19 (*n* = 56) were altered compared with age- and sex-matched healthy controls (*n* = 47), and were associated with clinical features and the gut microbiome composition [67]. A prospective study demonstrated that neuropsychiatric symptoms and fatigue in patients with long COVID correlated with nosocomial gut pathogens, including *Clostridium innocuum* and *Actinomyces naeslundii*, and that butyrate-producing bacteria showed a significant inverse correlation with post-acute COVID-19 syndrome [68]. Recently, Liu et al. [69] suggested that host phenotype and multi-kingdom microbiome profiling may be useful prognostic tools for COVID-19 patients.

Interestingly, high levels of fungal translocation from the gut and/or the lung epithelium were detected in the plasma of patients with long COVID compared with patients without long COVID [70]. Collectively, it is likely that alterations in the gut microbiome and microbes producing metabolites (e.g., butyrate) might contribute to neurological and psychiatric symptoms, as well as GI symptoms, in COVID-19 patients through the gut–brain axis (Figs. 1 and 3).

### Damaged intestinal barrier by SARS-CoV-2 infection

SARS-CoV-2 is known to bind to ACE2 (angiotensin-converting enzyme 2) [16, 71]. The intestinal tract with high expression of ACE2 is a major site of extrapulmonary infection of SARS-CoV-2 [72]. It is suggested that SARS-CoV-2 in the blood is a potential route of intestinal infection, and that virus is released into the gut and infects surrounding intestinal epithelial cells of the intestinal tract. Infection of SARS-CoV-2 in the GI tract can damage the intestinal barrier and intestinal–blood barrier, resulting in GI symptoms. Furthermore, prolonged intestinal infection of SARS-CoV-2 and damaged intestinal–blood barrier might cause persistent GI symptoms [72, 73]. Collectively, it is likely that abnormalities in gut–microbiome by damaged intestinal barrier could cause a number of psychiatric and neurological symptoms in COVID-19 survivors through the gut–brain axis (Figs. 1 and 3).

### Abnormalities in the nasal, oral, or lung microbiome in COVID-19 patients

Although the abundance of the nasal and oral microbiomes is lower than that of the gut microbiome, these microbes may contribute to smell and taste dysfunctions in patients with COVID-19. A recent study showed that reduced abundance of *Corynebacterium* in the nasal microbiome was associated with the loss of olfactory function in COVID-19 patients [74]. Haran et al. [75] demonstrated that COVID-19 patients with prolonged symptoms had a higher abundance of inflammation-causing oral microbes, and that the oral microbiome of patients with long COVID was similar to that of patients with chronic fatigue syndrome, suggesting an association between the oral microbiome and long COVID.

In addition, accumulating evidence shows that microbes in the lung are altered in a number of diseases, suggesting the role of the lung microbiome in health and disease [76]. We recently reported that antibiotic-induced microbiome depletion could attenuate lipopolysaccharide (LPS)-induced acute lung injury in mice, suggesting a role for the gut–lung axis in lung injury [77]. A recent study demonstrated that the lung microbiome could regulate autoimmune pathology in the rat brain, suggesting the existence of a lung–brain axis [78]. Taken together, it is possible that lung tissue damage and dysbiosis of the lung microbiome after SARS-CoV-2 infection may, in part, contribute to long COVID symptoms, including neurological and psychiatric symptoms, through the lung–brain axis. In addition to the gut microbiome, it is likely that nasal, oral, and lung microbes might modulate a number of long COVID symptoms through body–brain communication (Fig. 3).

### Dysbiosis of the gut microbiome after COVID-19 vaccination

COVID-19 vaccines play a central role in preventing serious illness, hospitalization, and death caused by SARS-CoV-2 infection. Ng et al. [79] investigated the gut microbiome composition of adults after injection with the inactivated vaccine (CoronaVac; Sinovac) or the mRNA vaccine (BNT162b2; BioNTech) and found that the immune response was significantly lower in recipients of the CoronaVac vaccine compared with the BNT162b2 vaccine. *Bifidobacterium adolescentis* was more abundant in the stool samples of individuals with high levels of neutralizing antibodies to CoronaVac vaccine. There was also a positive correlation between neutralizing antibodies in BNT162b2 vaccinees and the total abundance of microbiome possessing flagella and fimbriae. Interestingly, the abundance of *Prevotella copri* and two *Megamonas* species was enriched in stool samples from subjects with fewer adverse events following either of the two vaccines. This study suggests that specific gut microbiome markers may be associated with an improved immune response and a decrease in adverse events following COVID-19 vaccination [79].

Using metagenomic and targeted metabolomics analyses, Tang et al. [80] reported that BBIBP-CorV vaccination of Chinese adults (*n* = 207) resulted in an altered composition of gut microbiota and functional pathways, and that the gut microbiota and its functional profiles correlated with the response of vaccine. The levels of short-chain fatty acids (SCFAs) in stool and blood samples were increased in the high antibody response group compared with the low antibody response group. Furthermore, there was a positive correlation between several SCFAs and the antibody response. This study suggests that the gut microbiome and microbiome-producing SCFAs are associated with the BBIBP-CorV vaccine response [80].

### Treatment strategy based on the gut–microbiota–brain axis

#### Diet

Modification of the diet has been proposed to improve dysbiosis of the gut microbiome and clinical outcomes in COVID-19 patients including long COVID [58]. A prospective cohort study of participants (*n* = 592,571) demonstrated that dietary intake of healthy plant-based foods was associated with a lower risk and severity of COVID-19 [81]. A retrospective study in Taiwan (*n* = 509) showed a significant association between a vegetarian diet and a lower severity of symptoms in older patients with COVID-19, with older patients with a non-vegetarian diet showing a higher risk of severe symptoms [82]. Collectively, it is likely that plant-based foods may be prophylactic for acute and long COVID (Fig. 3).

#### Probiotics and prebiotics

Several retrospective clinical studies demonstrated that oral administration of probiotics and prebiotics could induce antiviral effects and have positive effects on the altered composition of the gut microbiome in patients with COVID-19, resulting in an improvement in clinical outcomes [58]. A randomized, quadruple-blinded, placebo-controlled trial showed that supplementation with probiotics for 30 days reduced the nasopharyngeal viral load, lung infiltrates, and the duration of both digestive and non-digestive symptoms in adult symptomatic outpatients with COVID-19 compared with the placebo [83]. A longitudinal cohort study using COVID-19 patients (*n* = 200) showed a negative correlation between the regular intake of yogurt containing probiotics and disease severity in patients with COVID-19 [84]. A recent meta-analysis of nine studies demonstrated that supplementation with probiotics was associated with a significant (51%) reduction in the severity of symptoms in COVID-19 patients [85]. Collectively, it is likely that supplementation with probiotics and prebiotics is beneficial for limiting the severity of symptoms in patients with acute and chronic COVID-19 (Fig. 3).

#### Short-chain fatty acids (SCFAs)

In addition to significant alterations in the gut microbiota, patients with severe/critical COVID-19 had reduced levels of SCFAs (acetic acid, propionic acid, butyric acid) produced by their gut microbiome [63]. It is noteworthy that the reduced levels of butyric acid in the feces samples from severe/critical patients persisted beyond 30 days after recovery [63], suggesting persistent reduced levels of butyric acid-producing microbes in the microbiome of patients with long COVID. As mentioned above, patients with long COVID also had reduced levels of anti-inflammatory butyrate-producing microbes in their microbiome [63]. Considering the role of microbiome-derived SCFAs (e.g., butyrate) in the gut–microbiota–brain axis [86, 87], it is possible that butyrate could be a potential therapeutic SCFA for long COVID (Fig. 3).

#### Fecal microbiota transplantation (FMT)

Fecal microbiota transplantation (FMT) is a method to restore the balance of the gut microbiome via the transfer of a healthy microbiome [58, 88]. Some case reports showed that FMT from healthy subjects improved GI symptoms, abnormal blood immunity markers, and dysbiosis in patients with COVID-19 [89], and attenuated the severe symptoms in patients [90]. Although FMT may be a potential therapeutic approach for long COVID, further studies are warranted to ensure the safety of FMT in this context (e.g., the risk of *Clostridioides difficile* infection) (Fig. 3).

#### Vagus nerve stimulation (VNS)

The vagus nerve is the main nerve of the parasympathetic division of the autonomic nervous system. Accumulating evidence shows that the vagus nerve plays a key role in communication between the gut and the brain [91–94]. For example, we previously reported that subdiaphragmatic vagotomy blocked the onset of a depression-like phenotype in mice after LPS administration [95], FMT from mice with depression-like phenotypes [96, 97], or in *Chrna7* knockout mice with depression-like phenotypes [98]. Furthermore, subdiaphragmatic vagotomy blocked demyelination in the brain after dietary intake of cuprizone-containing food [99]. Collectively, it appears that the vagus nerve plays a crucial role in communication between the periphery and the brain.

Vagus nerve stimulation (VNS) has been used in the treatment of refractory epilepsy and treatment-resistant depression. In 2000, Borovikova et al. [100] demonstrated that VNS could attenuate LPS-induced systemic inflammation in rodents, indicating the potent anti-inflammatory action of VNS [94]. Thus, it is likely that VNS can attenuate inflammation both in preclinical models and in humans through activation of cholinergic anti-inflammatory pathways [101]. A randomized clinical trial demonstrated that transcutaneous auricular VNS (90 min twice a day for consecutive 7 days) caused significant reduction of C-reactive protein, interleukin-6, and depression score in hospitalized patients with COVID-19 [102]. Given the crucial role of the vagus nerve in the gut–brain axis, the author proposes that VNS could be a potential therapeutic approach for long COVID through its potent anti-inflammatory activity (Fig. 3). Several clinical trials (NCT05608629, NCT05679505, NCT05630040, NCT05205577, NCT05225220, NCT05764070, NCT05445427) using VNS for long COVID are currently underway.

## Conclusion

Based on a number of clinical studies, COVID-19 survivors experience a number of long COVID symptoms, including many psychiatric and neurological symptoms. Although the detailed biological mechanisms underlying long COVID remain elusive, several hypotheses have been proposed [3]. First, as SARS-CoV-2 was detected in the brain of patients who died from COVID-19, it is possible that SARS-CoV-2 can cross the BBB after infection, resulting in neuroinflammation and subsequently a number of acute and chronic neurological and psychiatric symptoms. Second, EBV reactivation after SARS-CoV-2 infection could contribute to acute clinical symptoms and long COVID symptoms. The transcription factor XBP1 in the ER plays a role in the reactivation of EBV [44]. Given the interaction of XBP1 and sigma-1 receptor in the ER, it is likely that sigma-1 receptor agonists (i.e., fluvoxamine), which may block EBV reactivation, would be potential therapeutic drugs to limit clinical deterioration after infection and long COVID symptoms [51–53]. Finally, SARS-CoV-2 can enter in the GI tract and cause dysbiosis of the gut microbiota in patients with COVID-19. It is shown that infection of SARS-CoV-2 could damage the intestinal barrier, resulting in dysbiosis of gut microbiome and GI symptoms. Given the role of the gut–brain axis in systemic inflammation, it is possible that dysbiosis of the gut microbiome in COVID-19 survivors may induce neuroinflammation in the brain through the gut–brain axis. Gut microbiome-based approaches such as a plant-based diet, probiotics and prebiotics, microbiome-derived SCFAs, and VNS might be beneficial for a variety of long COVID symptoms. However, randomized control studies are warranted to ascertain the therapeutic potential of these strategies in the context of long COVID.
